# Coordinative variability and overuse injury

**DOI:** 10.1186/1758-2555-4-45

**Published:** 2012-11-27

**Authors:** Joseph Hamill, Christopher Palmer, Richard E A Van Emmerik

**Affiliations:** 1 Department of Kinesiology, Biomechanics Laboratory, University of Massachusetts Amherst, 30 Eastman Lane, Amherst, 01003, MA, USA

**Keywords:** Variability, Coordination, Dynamical systems, Overuse injury

## Abstract

Overuse injuries are generally defined as a repetitive micro-trauma to tissue. Many researchers have associated particular biomechanical parameters as an indicator of such injuries. However, while these parameters have been reported in single studies, in many instances, it has been difficult to verify these parameters as causative to the injury. We have investigated overuse injuries, such as patella-femoral pain syndrome, using a dynamical systems approach. Using such methods, the importance of the structure of coordinative variability (i.e. the variability of the interaction between segments or joints) becomes apparent. We view coordinative variability as functionally important to the movement and different from end-point or goal variability. Using concepts derived from the work of Bernstein, we conducted studies using a continuous relative phase and/or modified vector coding approaches to investigate the coordinative variability of overuse injuries. Consistently, we have found that the higher variability state of a coordinative structure is the healthy state while the lower variability state is the unhealthy or pathological state. It is clear that very high coordinative variability could also result in injury and that there must be a window of ‘higher variability’ in which non-injured athletes function. While this finding that coordinative variability is functional has been shown in several studies, it is still not clear if reduced variability contributes to or results from the injury. Studies are currently underway to determine the potential reasons for the reduced variability in injured athletes. Nevertheless, our laboratory believes that this understanding of how joints interact can be important in understanding overuse injuries.

## Introduction

The incidence of overuse injuries in running has not changed over the last 30 years [1]. The knee, leg and foot are the most frequently injured by runners with knee injuries reported by approximately 45% of runners. Running injuries are generally divided into two broad categories: 1) traumatic injuries; and 2) cumulative micro-trauma injuries. Traumatic, or acute, injuries can be thought to result from a single, large magnitude force that is usually applied over a very short period of time. For example, an Achilles tendon rupture is defined as a traumatic injury. Cumulative micro-trauma injuries, often called overuse or chronic injuries, result from a number of repeated low magnitude impacts applied over a considerable time period. Most running injuries fall into the category of overuse injuries. Examples include patellofemoral pain, Achilles tendinitis, and iliotibial band syndrome.

There have been many noted risk factors related to overuse injuries in running. Several risk factors often cited are: 1) repeated loading; 2) foot/ground contact force; 3) running footwear [2]; 4) running surfaces; 5) anatomical predisposition; 6) training errors; and 7) previous injury [2]. While there is a multiplicity of variables thought to be risk factors for overuse injuries, it is without question that some of the factors are biomechanically-related. A significant problem in studying overuse injuries is that there are multiple interactions among the risk factors making it difficult to determine the etiology of the injury. A related problem in determining the cause of an overuse injury is the general lack of prospective studies, which makes it difficult to draw causal inferences from retrospective data. Additionally, the use of the typical dependent measures and standard kinematic and kinetic analyses cannot lead to a definitive cause of injury.

Over the last 30–35 years, biomechanists have primarily used kinematic and kinetic analyses to probe the etiology of overuse injuries. Of particular interest has been the calculation of rearfoot angle (i.e. the motion of the calcaneus relative to the tibia in the frontal plane). “Excessive” rearfoot motion is often cited as a cause of overuse injury [3,4] although there is no clinical definition as to what is “excessive.” From a kinetic standpoint, ground reaction forces have often been used to relate external forces to the etiology of impact injuries [5,6]. The parameters that are often used in this type of analysis are the peak impact force and the loading rate. The peak impact force has not proven successful in differentiating loads on the body in individuals with differing injuries [7,8]. On the other hand, loading rate (i.e. the slope of the force-time curve from 20%-80% of the peak impact force) has shown some promise in differentiating healthy and injured groups [9,10]. Joint moments and forces, calculated from an inverse dynamics procedure, have also been used in injury research. For example, the knee adduction moment has been related to the incidence of patellofemoral pain (e.g. [11]).

For the most part, however, the traditional kinematic and kinetic analyses have provided definitive results in that they have distinguished between runners with and without injuries and between healthy and injury-prone individuals. The explicit cause of injury has not been forthcoming in these studies, and may not be empirically accessible given the interacting injury mechanisms involved. Thus, the results of these studies have not lead to a clearer understanding of the injury mechanisms and have not brought about a rehabilitative process for recovery or prevention from these injuries. For example, there are numerous studies on iliotibial band syndrome all of which present different distinguishing factors between those with and without iliotibial band syndrome [12,13]. Because there are many contributing factors to injury, the level of analysis “above” these interacting injury mechanisms may be fruitful for characterizing injury etiology. This macroscopic analysis of the combined contributions of interacting injury mechanisms to the state of a system (the states being injured, uninjured, progressing towards injury, or recovering from injury) underlies the Dynamic Systems approach, as it inherently recognizes that there may be many injury “mechanisms” interacting to cause such a state. Thus, it appears necessary to explore other than the traditional techniques to fully understand the mechanisms and etiology of injury to answer the questions that have posed previously. In this paper, we present evidence that segmental coordinative phase relations and coordinative variability can be helpful in determining overuse injuries and characterize the macroscopic level of analysis useful for determining injury etiology.

### The dynamical systems approach

Smooth goal directed movements require the integration and coordination of the individual degrees of freedom at different spatio-temporal scales (e.g., motor units, muscles, joints/segments) into functional units. According to Turvey [14], coordination involves bringing the multiple degrees of freedom at each level into proper relations. These proper relations are formed because of redundancy in the motor system. Many years ago, Bernstein described this redundancy in the available degrees of freedom and he strongly advocated that action systems with multiple degrees of freedom enable different solutions to a particular task [15,16]. Functional systems that are stable and adaptable use all their degrees of freedom effectively in order to optimize task performance [17]. There are components to analyzing a task, according to the Bernstein perspective, which are key [18]. First is that relationships between parts is critical and not an investigation of the parts themselves. This position derives from the fact that the many individual parts can be organized in a large number of ways to sub serve the same coordination pattern. The second key point is that variability is of paramount importance, as it provides metric related to the variety of ways in which the coordinative pattern is maintained.

### Types of variability

The traditional view of variability is based on the concept of ‘end-point’ variability. From this perspective, the variability of the product of a movement (e.g. stride length, stride time, etc.) should be less in a healthy individual and greater in a less healthy individual [19]. That is, expert performers would have less variability than novices and healthy individuals would have less variability than those with movement disorders. It is now clear, however, that stability in the performance of goal-directed performance (low variability at the ‘working-point’) is only achievable only through variability at the level of coordinative relations underlying that performance [15,20-22].

The view put forth in this paper shares this perspective that coordinative variability would in fact have the opposite interpretation of ‘end point’ variability, and that these two concepts of variability must be integrated in any functional movement analysis. To illustrate the difference, we will present a paper by Arutyunyan et al. [21] who conducted a pistol shooting test with experts and novices. They found that expert pistol shooters had less ‘end-point’ variability (i.e. the ability to hold the barrel of the pistol steady) than the novices. On the other hand, they reported that the coordinative variability between the shoulder, elbow and wrist of the expert shooters was greater than the novices. This study shows that the two types of variability are different, have different interpretations, and are related when goal-directed movements are examined. In gait dynamics, the goal-directed ‘end point’ is not a discrete spatial location, but the maintenance of segmental relations (co-ordination) over many cycles that define the locomotor pattern itself.

In most research in biomechanics and motor control, variability is traditionally equated with noise, considered detrimental to system performance and is typically eliminated from data as a source of error. Equipment noise, electrical interference and movement artifacts are examples of sources contributing to this measurement noise. A second source of biological variation is dynamical variability and arises from within the system to be studied. In this case no clear separation can be obtained between the ‘original’ signal and variability. This form of variability emerges from underlying nonlinearities and is important for pattern formation, sensation, and perception in biology [23].

Thus, variability observed in human performance can be fundamentally of two different forms, namely noise due to measurement error and coordinative variability or variation due to inherent dynamics of the system [24]. It has been suggested that coordinative variability is simply ‘noise’ in the system. According to Kantz and Schreiber [24], we can define a system as:

$$x_{n+1}=F\left(x_{n}\right)$$

Measurement noise is additive to the system:

$$x_{n+1}=F\left(x_{n}\right)+\eta_{n}$$

where η_n_ is the measurement noise. We have means such as filtering to eliminate this measurement noise because it is of sufficiently high frequency [25]. On the other hand, coordinative variability is a part of the higher order dynamic of the signal:

$$x_{n+1}=F\left(x_{n}+\beta_{n}\right)$$

where β_n_ is the coordinative variability. Coordinative variability cannot be removed from the signal. The multiple degrees of freedom involved in the coordination and control of human movement are a potential source of this dynamical variability, which is suggested to arise from the many combinations of interacting parts from which patterned movement emerges.

**Figure 1:**
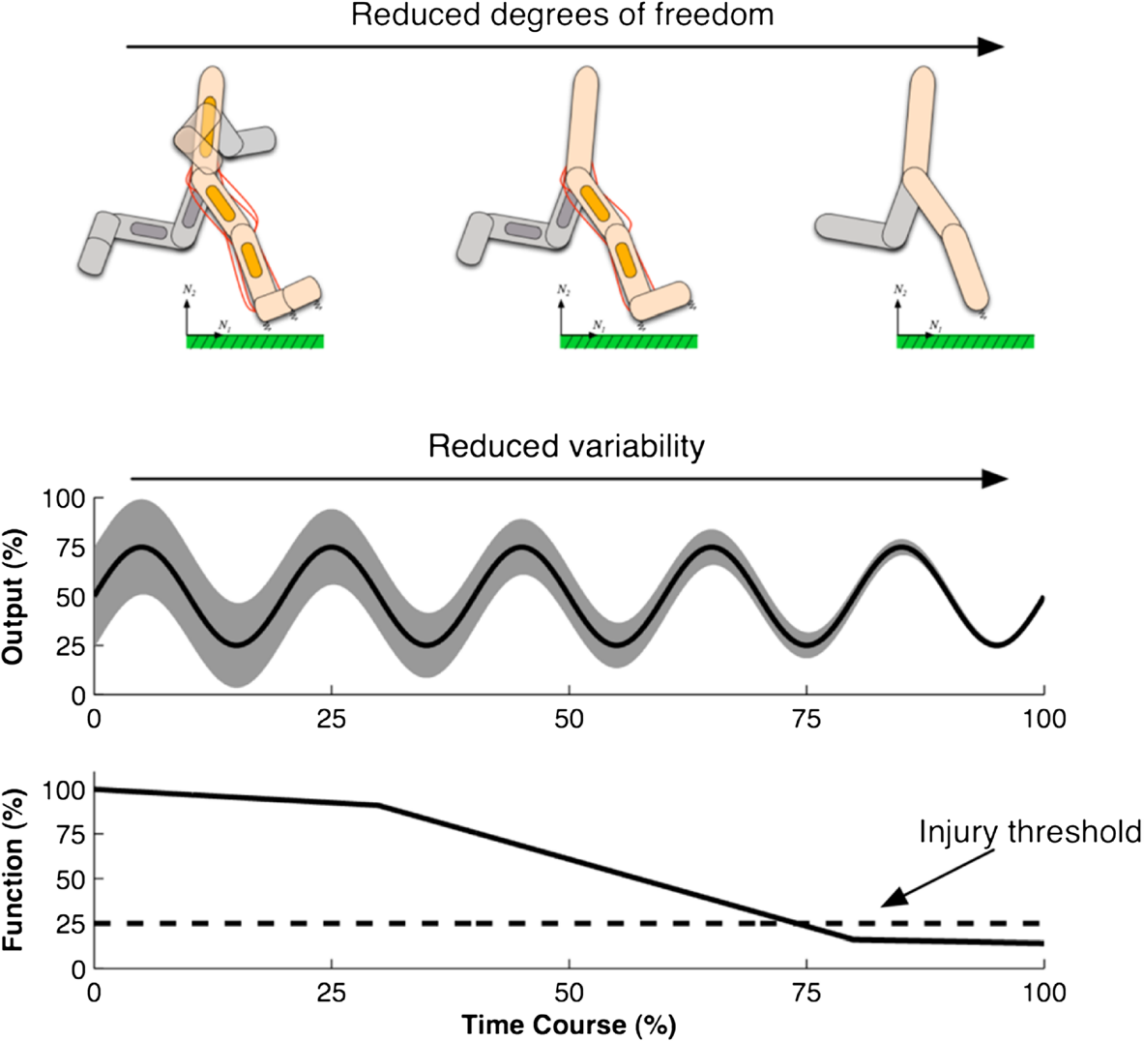
**Loss of complexity hypothesis based on the work of Lipsitz and colleagues (2002) applied to injury or pathology.** Top and middle panels: over time reductions in effective degrees of freedom, interacting components and synergies become associated with a loss of variability in the system. When these reductions in degrees of freedom and variability reach a critical threshold, injury or disease may emerge (bottom panel).

There is a growing body of literature in the biological and physical sciences stressing the beneficial and adaptive aspects of variability in system function. From this perspective, increased variability is no longer rigidly associated with decreased skill levels, injury and health. Instead, the path to frailty or injury is identified in this emerging perspective by a loss of variability in fundamental variables reflecting biological function [26]. This *loss of complexity hypothesis* can also be applied to neurological disease or orthopedic injuries (Figure 1). The proposed relation between loss of variability and loss of complexity has to do with the reduction in the many interacting degrees of freedom that underlie a macroscopic state of affairs (coordinative pattern relationships) in the system of interest. Over time, reductions in effective degrees of freedom, interacting components and synergies involved in the control of the biological system may become associated with a loss of variability. When these reductions in degrees of freedom and variability reach a critical threshold, injury or disease emerge.

**Figure 2:**
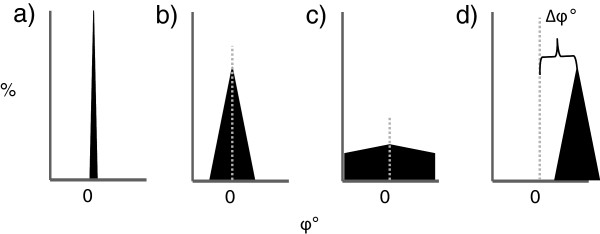
**a) Absolute coordination with Low coordinative variability; forces during locomotion are distributed over a smaller surface area and contribute to overuse injury. b**) Relative coordination with an optimal coordinative variability pattern, distributing forces across biological tissues in a manner that does not contribute to overuse injury with appropriate training cycles. Potential paths to injury via High coordinative variability include: **c**) maintenance of phase relations around similar values with increased variability beyond the norm; **d**) similar coordinative variability, but an offset from the normative phase relations generally observed (fixed point drift). While relations between a) and b) have been demonstrated in differentially injured participants, the generation of normative values and the potential etiology of injury involving both low and high coordinative variability require prospective studies within groups of interest.

An important emphasis in recent research in biomechanics is link between variability and overuse injuries [27], [28,29]. Even in repetitive activities such as running the motions of the body’s segments will vary somewhat, and these variations may be functional and healthy. Several studies have now demonstrated an association between reduced coordination variability and orthopedic disorders or overuse injuries e.g. [30]. The relationship between absolute and relative coordination and coordinative variability and how this relates to overuse injuries is presented in Figure 2. We propose that absolute coordination with its low variability causes forces to be distributed across small surface areas, possibly resulting in overuse injuries. In contrast, the variations present during relative coordination allow joint or tissue forces to be distributed, thereby minimizing the change for overuse injuries.

### Approaches to determining coordinative variability

In injury research, we often refer to the concept of coupling. Coupling in this context refers to the interaction between segments or joints and implies that the motion of one segment (or joint) can influence the motion of another segment (or joint). For example, in the lower extremity, the motion of sub-talar joint eversion must be accompanied by internal tibial rotation and external femoral rotation. Also, sub-talar joint inversion must be accompanied by external tibial rotation and internal femoral rotation. The motions of these segments are said to be coupled and deviations from these motions are referred to as “asynchronous” and were thought to have implications for injury.

The three primary methods [31] that evaluate the coordination and coordination variability of coupling behaviors are: 1) discrete relative phase (evaluates the timing of key events in each of the angle profiles); 2) vector coding (a spatial measure based on an angle-angle plot); and 3) continuous relative phase (a spatio-temporal measure based on the phase planes generated from the angular position and angular velocity of the segments). Each of these techniques has been used to assess coordination in injury research studies. There is no one right technique to assess coordination variability because the choice of the technique to use should be based on the question asked in the study.

#### Discrete relative phase

**Figure 3:**
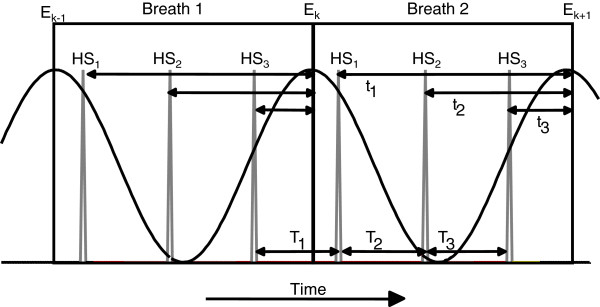
An example of a Discrete Relative Phase setup during running with two time-series: a) breathing inspiration-expiration (black solid line); and b) heel contact (gray solid line).

Discrete relative phase (DRP) illustrates a temporal phase relationship in a specific coupling. A discrete relative phase angle is determined at a discrete event during a movement cycle using the time-series profiles of two joint or segment angles or two other related physiological parameters. For example, many researchers have investigated the relative timing of knee flexion/extension and subtalar inversion/eversion during the support phase of a running stride [32,33]. The key event in this analysis would relate to the functionally important instance when the knee joint reaches maximum flexion. At this point in time, the subtalar joint should have reached maximum eversion. The initial point in the analysis is determined by another key event such as foot touchdown establishing time zero from which the other events are determined. The DRP angle (φ) is then calculated as follows:

$$\varphi =\frac{t_{1}-t_{2}}{T}\times 360{}^{\circ}$$

where t_1_ is the time to maximum knee flexion, t_2_ is the time to maximum subtalar eversion and T is the support period. The DRP angle can range from 0^o^ to 360^o^ where φ = 0^o^ or 360^o^ implies that the timing of the events are perfectly in-phase (i.e. occur at exactly the same instant in time). DRP angles between 0^o^ and 360^o^ indicate that the timing of the events are out-of-phase (i.e. one event lags behind the other event). To calculate the mean and standard deviation of the DRP angle over a number of trials (or footfalls in this example) circular statistics must be used. Another example of DRP is presented in Figure 3 in which breathing inspiration/expiration is coupled with stride frequency [34].

#### Modified vector coding

**Figure 4:**
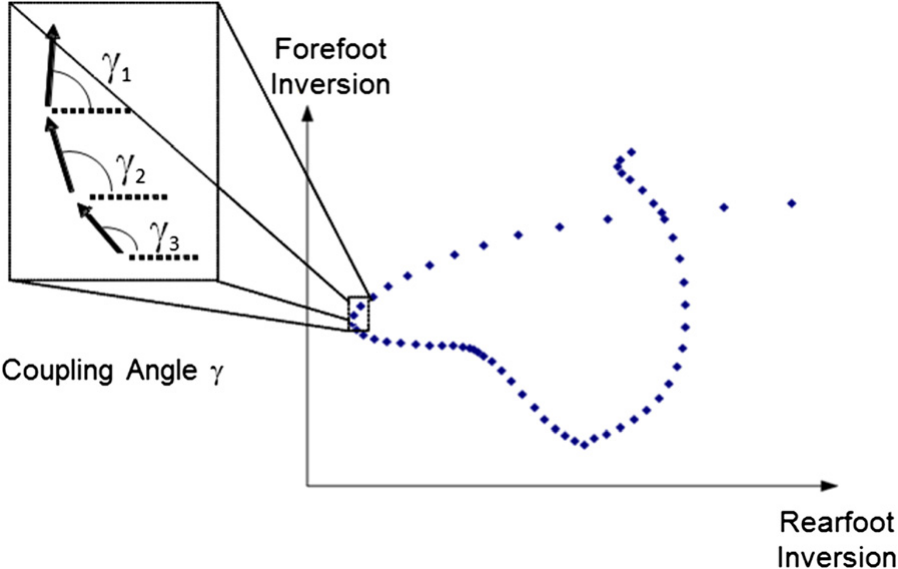
**Calculation of phase angles between rearfoot and forefoot inversion using a modified vector coding technique.** The phase angle is calculated relative to the right horizontal for each pair of contiguous points and then plotted across the time interval.

The modified vector coding approach is an adaptation of a method suggested by Sparrow et al. [35]. In this approach, a measure of coordination and thus coordination variability is assessed using angle-angle plots (see Figure 4). The orientation of a vector between two adjacent points on the angle-angle plot relative to the right horizontal is referred to as the coordination angle (φ). The resulting angles range from 0^o^ to 360^o^ where values of 0^o^, 90^o^, 180^o^ and 270^o^ indicate movement of one of the joints or segments. When the more distal segment is fixed and the proximal segment or joint is rotating the coordination angle is 0^o^ or 180^o^ while 90^o^ and 270^o^ indicate the opposite actions. The two segments or joint will move in the same direction with values of 45^o^ and 225^o^ while at 135^o^ and 315^o^ indicate equal movement but in opposite directions.

In this approach, couplings are determined that are relevant to the movement in question. The angles in the analysis are derived from standard 3-D kinematic procedures and are time-scaled to 100% of the cycle. This computation is done over many cycles (i.e. strides of gait) for each subject in each condition. Because the coordination angle is classified as circular variable, circular statistics must be performed to calculate the mean and standard deviation of multiple cycles [36].

#### Continuous relative phase

**Figure 5:**
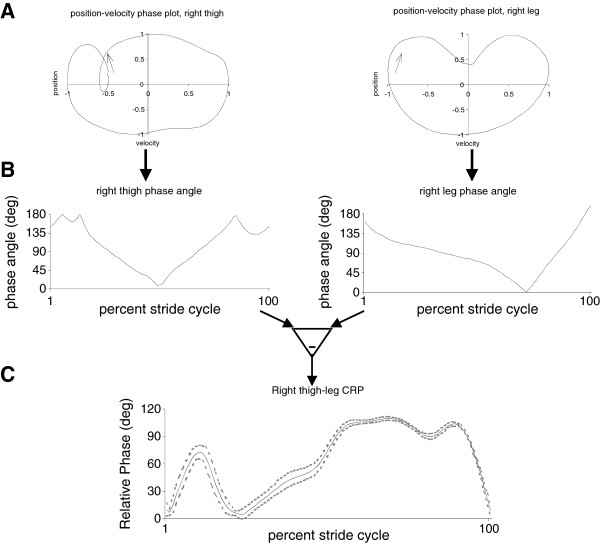
Calculation of a continuous relative phase angle: a) a phase plane constructed from a normalized position-velocity graph is developed for each segment or joint: b) phase angles are then calculated from each phase plane; c) a continuous relative phase angle is calculated by subtracting the two phase angles at each instant in time.

Continuous Relative Phase (CRP) is another measure of coordination from which we can develop a coordination variability profile. The CRP for a single stride or cycle is obtained by calculating the four-quadrant arctangent phase angle from a parametric phase plot (position vs. velocity) of the segments or joint of interest. For each of the time-series angles of one segment or joint, the normalized angle is plotted against the normalized velocity. The normalization procedure is a critical step [37]. Generally, each of the time-series profiles are time normalized such that a cycle ranges from 0–100%. At this point, a phase angle between adjacent points on the position-velocity phase plane is calculated for each instant in time across the cycle (see Figure 5a). The CRP angle is found by subtracting the phase angle of one segment or joint from the other at each point in time over the entire cycle (see Figure 5b):

$$CRP\left(t\right)=\varphi_{1}\left(t\right)-\varphi_{2}\left(t\right)$$

where φ_1_ (t) and φ_2_ (t) are the normalized phase angles for segment/joint 1 and segment/joint 2 respectively. CRP angles can range from 0^o^ to 360^o^ but there is a redundancy in certain angles and the scale is usually presented as 0^o^ to 180^o^. The assumption made here is that CRP(t) = 0^o^ indicates that the respective segments are moving in-phase while a CRP(t) = 180^o^ indicates the segments/joints are anti-phase. Any angle between these extremes indicates a relative amount of in- or anti-phase.

An ensemble profile can be calculated by averaging on a point-by-point basis across multiple cycles. CRP variability (i.e. coordination variability) may be calculated as the standard deviation on a point-by-point basis over the complete cycle (see Figure 5c), or over a portion of the movement pattern of functional interest to the research questions (e.g. mid-stance phase only).

### The functional role of coordinative variability

Several studies in motor control and biomechanics have illustrated that coordinative variability has a functional role. It has been shown that variability is important for coordinative changes in bimanual coordination and in gait [38-41]. The hypothesis put forward by Lipsitz [26], referred to as the ‘loss of complexity hypothesis’, suggested that a lack of variability may be a characteristic of dysfunction in a performance, frailty or disease (see Figure 1).

**Figure 6:**
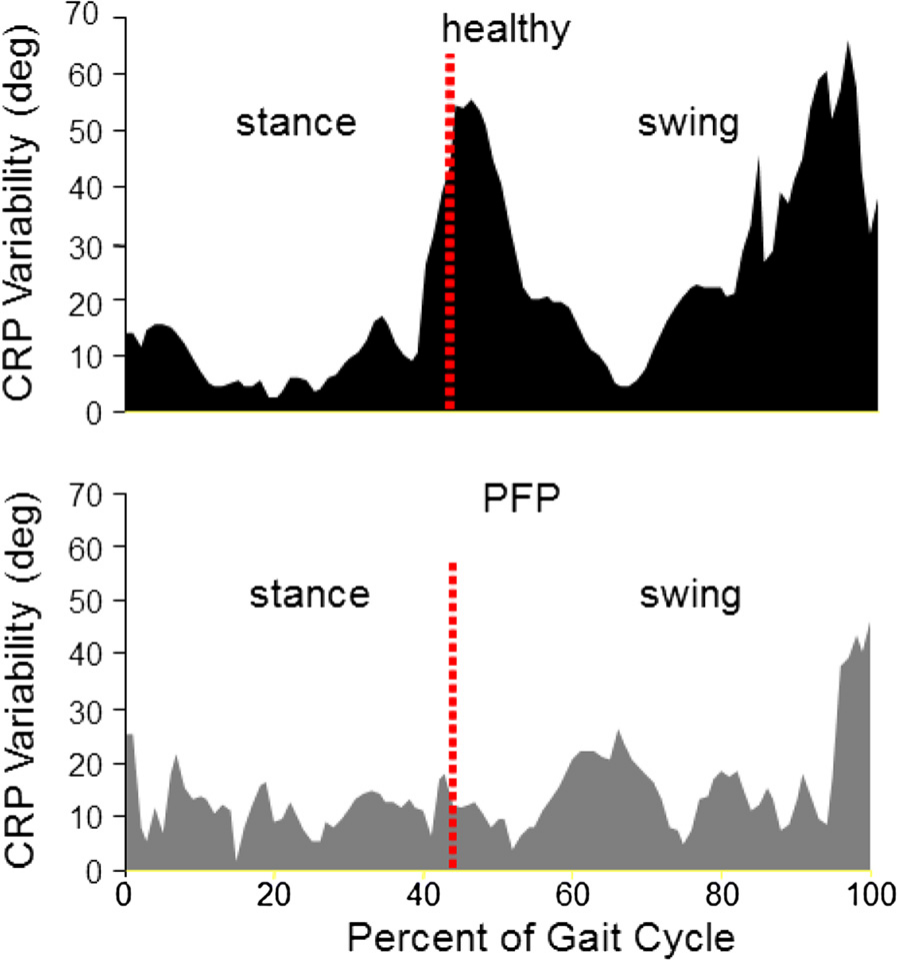
**Continuous relative phase (coordinative) variability during a stride in healthy and patellofemoral pain participants.** The coordinative variability is greater in the patellofemoral pain participants relative to the healthy participants. Figure adapted from Hamill et al. [27].

We emphasized the functional role of coordinative variability and related it to overuse injury using a dynamical systems perspective [27]. In this study, we assessed coordinative variability in individuals with and without knee pain. It was reported that greater coordinative variability (i.e. looser coupling between selected segments and/or joint) is the norm for a healthy individual. On the other hand, lower coordinative variability (i.e. tighter coupling) is the norm for individuals with knee pain (see Figure 6). This concept has been the focus of our research on overuse injuries since then.

The mechanism that we proposed suggested that there were numerous combinations of intra-segment coordination that could be accomplished by a healthy individual thus giving that individual the potential for higher coordinative variability (relative coordination patterns, Figure 3). However, in an injured individual, the number of combinations is reduced and thus the coordinative variability is significantly reduced. We have suggested that there is a threshold of coordinative variability below which an individual would be injured, and that coordinative variability may be used clinically to track the progression towards recovery [42,43].

Seay et al. [43] demonstrated that coordinative variability measures are able to discriminate between runners with low back pain, those recovered from low back pain, and those who never experienced low back pain. In this study, coordinative variability of trunk-pelvis transverse plane relations were greatest in those never injured, smallest in those with back pain, and in between these values for those who had ‘recovered’ from injury. This finding has two important implications: that coordinative variability is able to differentiate these stages of recovery from injury within a cross-sectional population, and; that despite being pain free, the ‘recovered’ runners still had lower coordinative variability than those never injured. This reduced variability in the pain free runners with previous injury compared to those never injured is thought to increase the stress on a smaller cross-section of soft tissues, contributing to the cyclic injury occurrence in low back pain and other chronic injuries. These types of findings suggest that longitudinal research using coordinative variability may be a fruitful next step to understanding the etiology of injury, and can help determine the progress of recovery from or progression towards and injured state.

### Functional coordinative patterns

**Figure 7:**
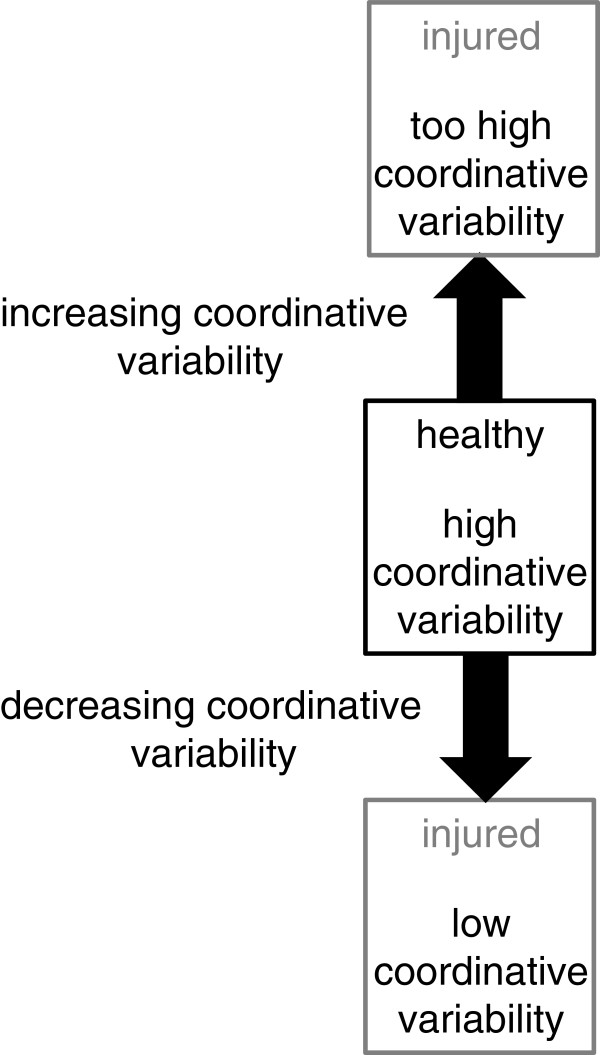
A schematic illustrating the relationship between high and low variability and injury.

Although Heiderscheit et al. [44] showed that the coupling angles were not substantially different between the PFP and healthy control subjects, this study revealed reduced joint coordination variability at heel strike in the PFP group. The variability of coordination can then be computed over many stride cycles using all of the coordination calculation techniques. It has been reported that the greater the coordination variability, the healthier the state of the system while lower variability has been related to a pathological or an injured system [27]. However, too much variability in a system may also be indicative of an injured individual (see Figure 7). That is, there is some window of variability in which a healthy individual functions. In the low variability state (i.e. the state in which an injured individual operates), it has been suggested that a reduced number of movements between the coupled joints or segments are available that may result in overuse of particular tissues causing an exacerbation to the injury. In addition, by reducing the number of available movement patterns, a less flexible system results that may not respond appropriately to an external perturbation. These findings have been substantiated in several studies on a variety of overuse injuries (e.g. [44]). In a study on tibial stress fractures in female runners compared to healthy, matched controls [45], the coordination variability in the injured limb was significantly less than in the non-injured limb while there was no difference in the level of variability in the limbs of the control subjects.

## Conclusions

Biomechanists have long used kinematic and kinetic analyses to investigate the etiology of running injuries. These analyses have provided definitive results in distinguishing between runners with and without injuries and between healthy and injury-prone individuals. However, these studies have not lead to a clearer understanding of the injury mechanism and have really not provided a rehabilitative measure that captures recovery from injury or prevention of the injury. If differences between groups with and without injuries are suspected, it is incumbent upon the researcher to use other methods to investigate the injury mechanisms in relation to the functional movement pattern of interest. Three methods that have been applied to clinical questions were presented in this paper, and have successfully discriminated between recovery stages from injury [43]. These methods illustrate differences that may give the researcher insight into the etiology of an injury as well as measures to assess progression towards potential injury (reduced coordinative variability with time vs. maintenance of ‘optimal’ coordinative variability over time). Even when the etiology of an injury can be determined from the traditional methods, the methods such as those suggested in this paper may still provide a relevant measure to help clinicians track the progression of recovery, assess differences in rehabilitative methods, or progression towards an injured state before injury occurs.

## Authors’ contribution

Each author contributed equally to the writing of this paper. All authors read and approved the final manuscript.

## Competing of interest

The authors declare that they have no competing interests.
